# Uncovering and classifying the role of driven nodes in control of complex networks

**DOI:** 10.1038/s41598-021-88295-4

**Published:** 2021-05-05

**Authors:** Yuma Shinzawa, Tatsuya Akutsu, Jose C. Nacher

**Affiliations:** 1 Department of Information Science, Faculty of Science, Toho University, Funabashi, Japan; 2 Bioinformatics Center, Institute for Chemical Research, Kyoto University, Uji, Japan

**Keywords:** Complex networks, Network topology

## Abstract

The widely used Maximum Matching (MM) method identifies the minimum driver nodes set to control biological and technological systems. Nevertheless, it is assumed in the MM approach that one driver node can send control signal to multiple target nodes, which might not be appropriate in certain complex networks. A recent work introduced a constraint that one driver node can control one target node, and proposed a method to identify the minimum target nodes set under such a constraint. We refer such target nodes to *driven nodes*. However, the driven nodes may not be uniquely determined. Here, we develop a novel algorithm to classify driven nodes in control categories. Our computational analysis on a large number of biological networks indicates that the number of driven nodes is considerably larger than the number of driver nodes, not only in all examined complete plant metabolic networks but also in several key human pathways, which firstly demonstrate the importance of use of driven nodes in analysis of real-world networks.

## Introduction

Recently, controllability approaches have been suggested to address control in complex networks^1,2^ and have also been widely used to study many real- world problems from cell biology to technological systems^3–14^. In particular, Liu et al. studied the minimum set of driver nodes in the context of structural controllability of linear complex networks^1^. One of their main results was that the minimum set of driver nodes can be computed in polynomial time using a reduction to the maximum matching (MM) problem on bipartite graphs. Structural controllability states the fundamental conditions such that a system can become controllable^1,15^. In the context of linear time-invariant dynamics, a system can be defined by the following equation:

$$\frac{{d \, \vec{x}(t)}}{dt} = A\vec{x} (t) + B\vec{u}(t)$$

where the $N \times N$ matrix ***A*** elements are the interacting parameter weights between *N* nodes. In other words, it determines the network of interactions between the system elements. The vector $\vec{x}(t) = (x_{1} (t),...,x_{N} (t))^{T}$ describes the state of the system with *N* nodes. An input $N \times M$ matrix ***B*** determines the coupling between the external controller (driver node) and the *controlled* node (driven node), where $M \le N$. Finally, the time-dependent input vector $\vec{u}(t) = (u_{1} (t),...,u_{M} (t))^{T}$ is used to control the system. These nodes are called *driver nodes*. As a result, the system is said to be controllable “*if it can be driven from any initial state to any desired final state in finite time*”. Each row of *B* contains at most one non-zero element, which means that each node receives signal from at most one driver node.

Hence, matrices ***A*** and ***B*** are defined to be structured matrices such that their elements are either fixed zeros (absence of interactions) or independent free parameters (weights). Lin stated that the system (***A***, ***B***) is structurally controllable if it is possible to choose the free parameters in ***A, B*** such that the system ***(A, B)*** is controllable in the traditional notion, i.e. the system satisfies the Kalman’s rank condition (rank ***C*** = *N*)^1,15^, where the matrix ***C*** is defined as ***C = (B, AB, A***^**2**^***B,…, A***^***N−1***^*** B).*** This means that if the system is controllable for a given set of parameters, then its controllability holds for almost any other parameter set^15,16^. Structural controllability is, therefore, very important because it enables us to determine controllability for systems whose interacting weights are typically unknown, such as most complex biological systems. Liu et al. demonstrated that it is possible to by-pass the computation of the rank ***C***, by computing instead the maximum matching in the network, which determines the minimum number of inputs or driver nodes needed to achieve control of the network.

Several works have also explored the maximum matching approach in network controllability and categorized the types of controls into those caused by *source nodes*, *external dilations* and *internal dilations*, and by using these metrics could classify the real network into several control profiles^17,18^.

The maximum matching (MM) approach works well in many cases but has some drawbacks when controlling real systems. The MM method assumes that, under certain conditions, one driver node can send control signal to many other target nodes^1^. We refer such target nodes to *driven nodes*. This assumption may not be practical in certain kinds of complex systems, especially in the control of biological networks. To be precise, it may lead to predict a different number of driver nodes and driven nodes in real-world networks.

It is worth noting that our definition of a driven node is based on the work by Pequito et al. which provided an algorithm to determine the minimum number of driver nodes under the condition that each driver node can send signal to exactly one node^19^. We also refer such nodes (that receive signals) as driven nodes. Therefore, this suggests that the MM-based algorithm by Liu et al. does not give the minimum number of driven nodes in the above sense.

However, analyses on large-scale real-world networks have not been done before to examine, quantify and compare both the numbers of driver nodes and driven nodes. Note that while Liu et al. analysed the number of driver nodes in many real-world networks, real data analyses were not compared to those computed using single-signal driver node constraint as discussed above.

The main contributions of this paper are as follows:

1. Compare the minimum number of driver nodes according to the MM method and that of driven nodes in large scale biological networks using techniques developed by Pequito et al. that determine the number of driver nodes under the condition that each driver node can send signal to exactly one node^19^. To be precise, we quantify the difference between the number of driver nodes and that of driven nodes in real-world networks for the first time through extensive computational experiments using a large number of biological networks, including human metabolic pathways and plant metabolic networks classified into four major lineages. The results indicate that the number of driven nodes is considerably larger than the number of driver nodes, not only in all examined complete plant metabolic networks but also in several key human pathways.
2. Develop a novel method to classify nodes into critical, intermittent, redundant categories for driven nodes. The method was obtained by combining knowledge from previous works^19,20^. It is well-known that the minimum set of driver nodes is not necessarily unique and there may be multiple solutions^1–21^. Similar problem exists in the identification of driven nodes. To address this issue, we develop a new algorithm that efficiently identifies and classifies all nodes uniquely into three control categories, namely critical, intermittent and redundant nodes. Critical driven nodes appear in all possible solutions. Intermittent driven nodes are included in at least one solution. Redundant nodes are those nodes that do not appear in any solution. We also utilize this novel algorithm to further clarify the differences between driver nodes and driven nodes in real-world networks. The results again suggest that driven nodes are more suitable for analysing biological networks than driver nodes.

It is worth noting that some works have characterized networks using a minimum control topology (MCT), in which the nodes and edges are classified terms of the fraction of nodes and edges that are always, sometimes, or never a part of an MCT^22^. However, there is no algorithm that identifies control categories for driven nodes.

**Figure 1:**
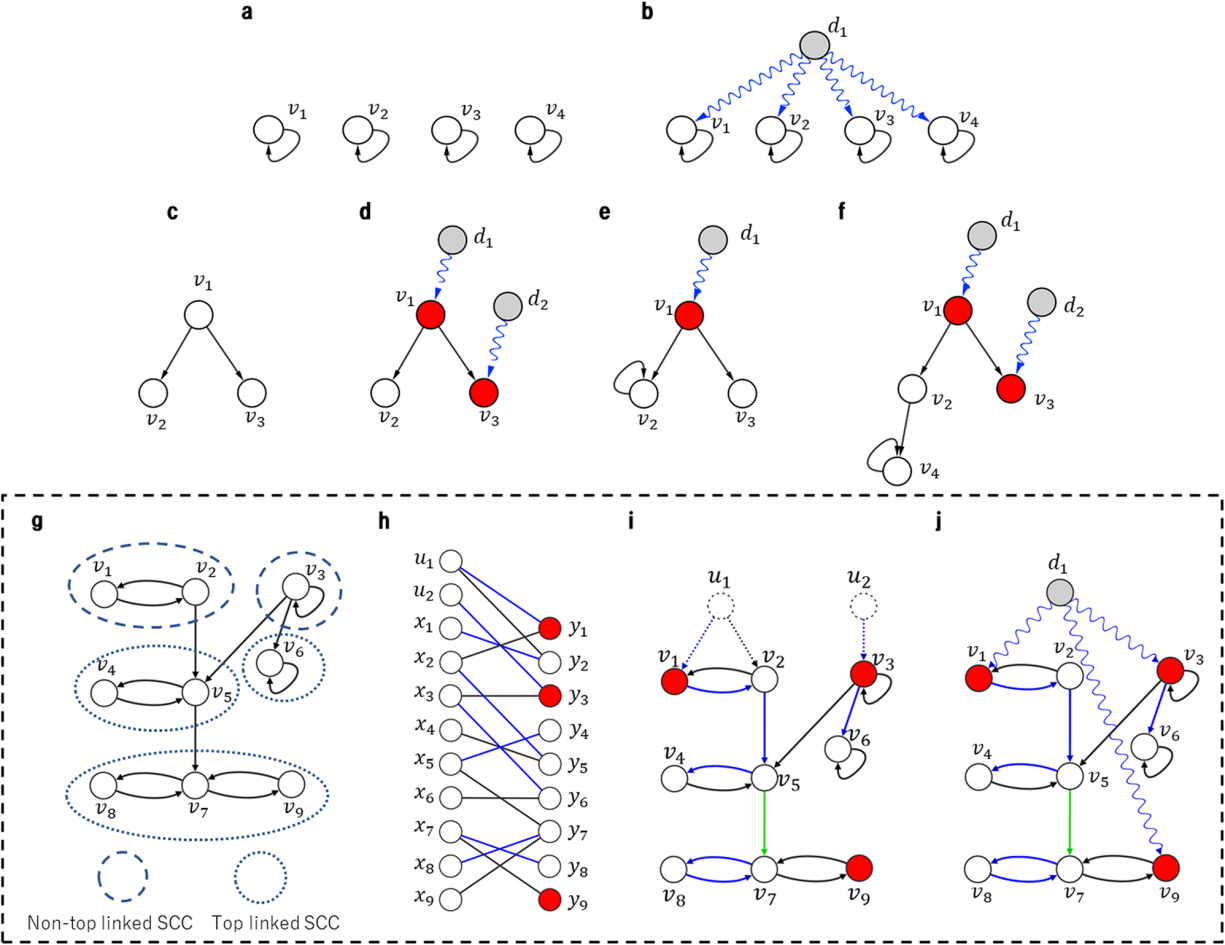
(**a**) Example of an original network with loops. (**b**) Using the MM theory, the entire network can be controlled by a single driver node (*d*_*1*_). Outgoing control signals are denoted in wavy blue arrows. (**c**) Example of a network without loops. (**d**) The network is controlled by driver nodes *d*_*1*_ and *d*_*2*_). (**e**–**f**) By adding a loop, the number of driver nodes decreases. (g) Example of an original network *G(V,E).* SCC denotes *Strongly Connected Component*. (**h**) Bipartite Graph (*V*_*L*_*, V*_*R*_*, E*_*B*_) and its bipartite matching (blue edges). (**i**) Stems and a bud obtained from the matching. The green edge (*v*_*5*_, *v*_*7*_) does not correspond to a matching but is included in a bud. (**j**) A single multi-signal driver node *d*_*1*_ controls three driven nodes (red). The computation of the MM may lead to a number of driver nodes that is smaller than that of the driven nodes.

Consider the network shown in Fig. 1a. Following the MM approach, it is sufficient to add one driver node *d*_*1*_, as shown in Fig. 1b. However, we need connections from *d*_*1*_ to all original nodes (i.e., *v*_*1*_, …, *v*_*4*_), which means that *d*_*1*_ should send control signals to all nodes. This explains the *theoretical* existence of “super-driver” nodes or “super-drugs”, i.e., a node (drug) that controls (binds) to all or multiple nodes (proteins). However, this type of multi-signal driver (*d*_*m*_) node is not plausible as a drug because it would result in many undesired side effects. Although this issue was also observed by Cowan et al.^16^, it has not received sufficient attention by the research community. However, the importance of self-loops and cycles to determine control profiles of profiles of complex networks was investigated in several works^17,18^.

To cope with this issue, a recent work introduced a constraint that one driver node can control one target node (i.e. driven nodes), and proposed a polynomial-time algorithm to identify the minimum number of target nodes under such a constraint^19^. The importance of the minimization of driven nodes was also highlighted in a recent review^23^. We followed their work^19^ to develop a novel algorithm to identify critical driven nodes in the real-worlds networks. Note that for downstream control, in the case that there are linear chains, downstream signals are not necessary. As shown in Fig. 1d–f, in some cases it is necessary if the downstream becomes a branch.

To efficiently control networks, the existence of loops and their positions in the network play key roles in both the minimum driver set problem and the minimum driven node set problem. Note that several works have shown the importance of self-loops and cycles to control a network in the context of the MM approach^17,18^. In particular, these features are important to determine the control profiles of complex networks. However, these works do not provide a technical or algorithmic method to determine or minimize the number of driven nodes in a network. On the other hand, and as stated above, Pequito et al. proposed a theoretical method for this calculation^19^. The network in Fig. 1c can be controlled using two driver nodes *d*_*1*_ and *d*_*2*_ with nodes *v*_*1*_ and *v*_*3*_ as driven nodes (Fig. 1d). By adding a single loop in node *v*_*2*_, both the driven set and driver set sizes are decreased to 1 (Fig. 1e). Furthermore, the addition of a single edge increases again the size of the driven and driver sets (Fig. 1f).

Indeed, a loop is closely related to the concept of the cactus in the structural controllability theory of Lin^15^. A subgraph consisting of an incoming edge and a directed loop is called a bud. Then, a cactus is a connected subgraph composed of a directed path (called a stem) plus buds, where the root of each bud must be a node in a stem or a node in a loop in another bud. The impact of stems and cycles on minimum control structures has been discussed^19^. Notably, Czeizler et al. also studied the minimum driven set problem^24,25^. However, their models are different from ours: they considered the target controllability problem. Indeed, in their model, both the minimum driver set problem and the minimum driven set problem are NP-hard. The difference between driver nodes^15^ and driven nodes^19^ rises when there are multiple cacti beginning with loops. These cacti can be controlled by using a single driver node^15^. Each cactus should have one driven node^19^ (or, equivalently, in the case that one driver node can directly control only one driven node, which is equivalent to the case that each column of *B* has at most one non-zero element).

To illustrate the mathematical problem, we set up a small synthetic network (Fig. 1g). The minimum weight maximum bipartite matching can be computed in polynomial time using Hungarian algorithm among others^19^. However, for ease of implementation, we describe an Integer Linear Programming (ILP) based method in the Supplementary Information file. The algorithm identifies three driven nodes (red) controlled by only one driver node *d*_*1*_ (Fig. 1h–j and Supplementary Fig. S1 online). This example shows that as long as there are many cycles in a network, the number of driven nodes tends to be much larger than the number of driver nodes. However, the driven nodes method was not applied to the analysis of large-scale real world networks.

The structure of the paper is as follows. In this study, we compiled data corresponding to a set of 84 human metabolic pathways and another large set of 70 plant metabolic networks. We then compute the numbers of driven and driver nodes and examine the differences. Next, we apply the developed new algorithm (see “Methods” section) to identify control categories for driven nodes and discuss the results.

## Results

### Analysis of driven nodes in human metabolic pathways

**Figure 2:**
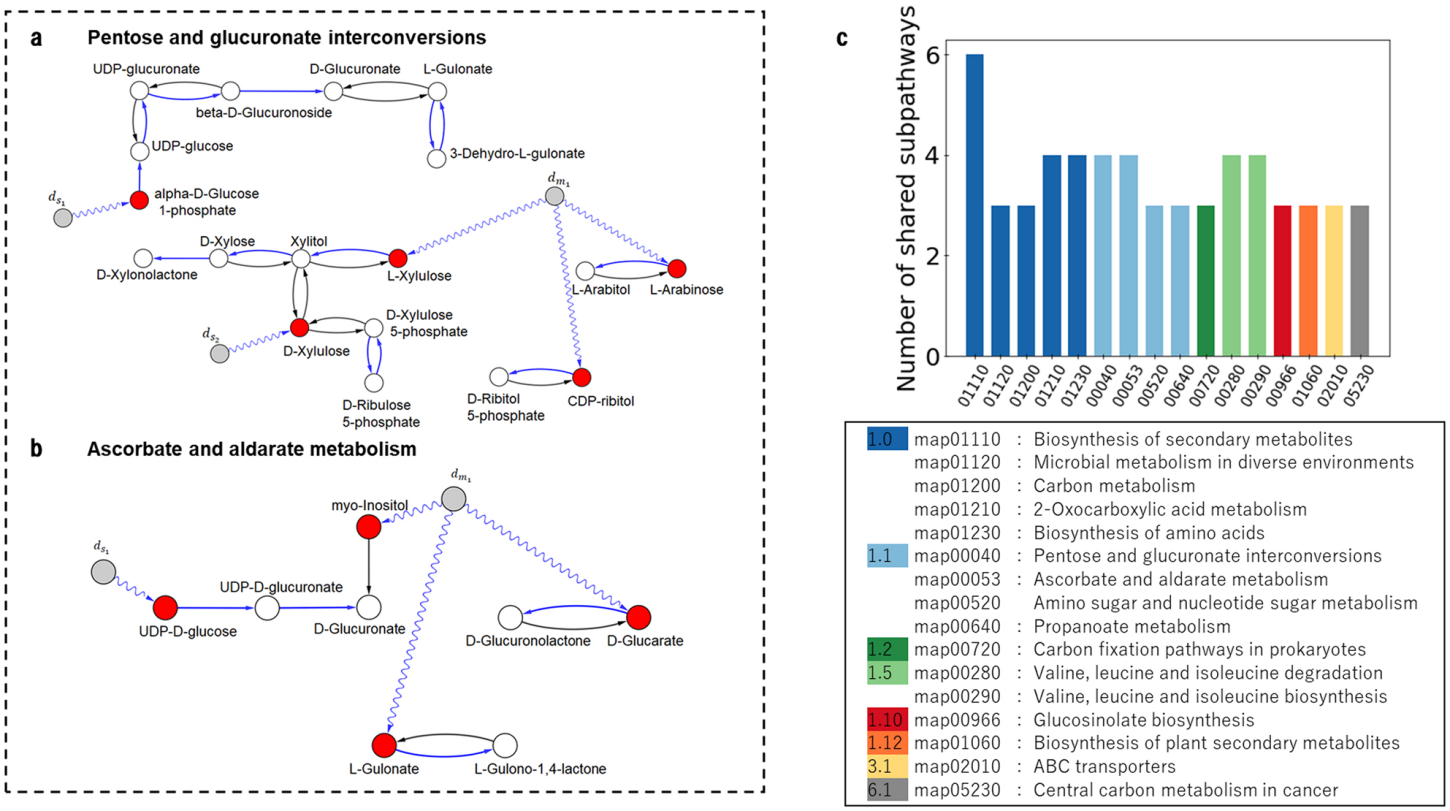
(**a**) The pentose and glucuronate interconversion pathway is controlled by three nodes: two single-signal driver nodes *d*_*s*_ and one multi-signal driver node *d*_*m*_ (grey nodes). There are five driven nodes denoted in red. |driven|-|driver| = 5–3 = 2. (**b**) The ascorbate and aldarate metabolism is controlled by one single-signal driver node *d*_*s*_ and by one multi-signal driver node *d*_*m*_ (grey nodes). There are four driven nodes denoted in red. |driven|-|driver| = 4–2 = 2. (**c**) The identified 24 chemical compounds driven by *d*_*m*_ nodes (drivers that send more than one outgoing signal) participate in many other functional pathways in the human metabolism, including disease pathways such as central carbon metabolism in cancer. Metabolic maps IDs correspond to the KEGG database metabolic pathway annotation.

To demonstrate that the computation of the MM leads to a number of driver nodes that is smaller than that of the driven nodes in natural systems, we collected a large number of directed biological networks, including many human metabolic pathways and 70 plant metabolic networks. First, a total of 84 human metabolic pathways, organized into 11 main metabolic functional pathways, were downloaded from the KEGG database^26^ (see SI Table S1 Excel file). The metabolic pathways are bipartite networks. Therefore, in this analysis, a total of 165 chemical compound and chemical reaction-centric unipartite networks were constructed and analysed independently. We then applied the proposed ILP for minimum weight maximum matching algorithm to identify the number of driven nodes in each pathway (see “Methods” section). The number of driver nodes was also calculated for comparison. The results show that at least 18 chemical compound networks (green) and 6 chemical reaction pathways (yellow) revealed a non-zero difference between the number of driver and driven nodes (see Table 1). This result suggests that at least one driver node sends more than one signal to control more than one driven node (chemical compound or reaction), mostly because of the existence of loops or cycles in the network. As an example, Fig. 2a shows the pentose and glucuronate interconversion pathway. This pathway is controlled by three nodes: two single-signal driver nodes *d*_*s*_ and one multi-signal driver node *d*_*m*_ (grey nodes). However, five driven nodes are identified (red nodes). Therefore, there is a difference between the number of driven and driven nodes. Another example shows the ascorbate and aldarate metabolism in Fig. 2b. This pathway is controlled by one single-signal driver node *d*_*s*_ and by one multi-signal driver node *d*_*m*_ (grey nodes). Four driven nodes (4) are identified (red nodes). Therefore, the number of driver nodes is smaller than that of the driven nodes, supporting our main hypothesis. As shown in Table 1, carbohydrate metabolism shows a large concentration of pathways in which the identified driven chemical compounds are located.

**Table 1:**
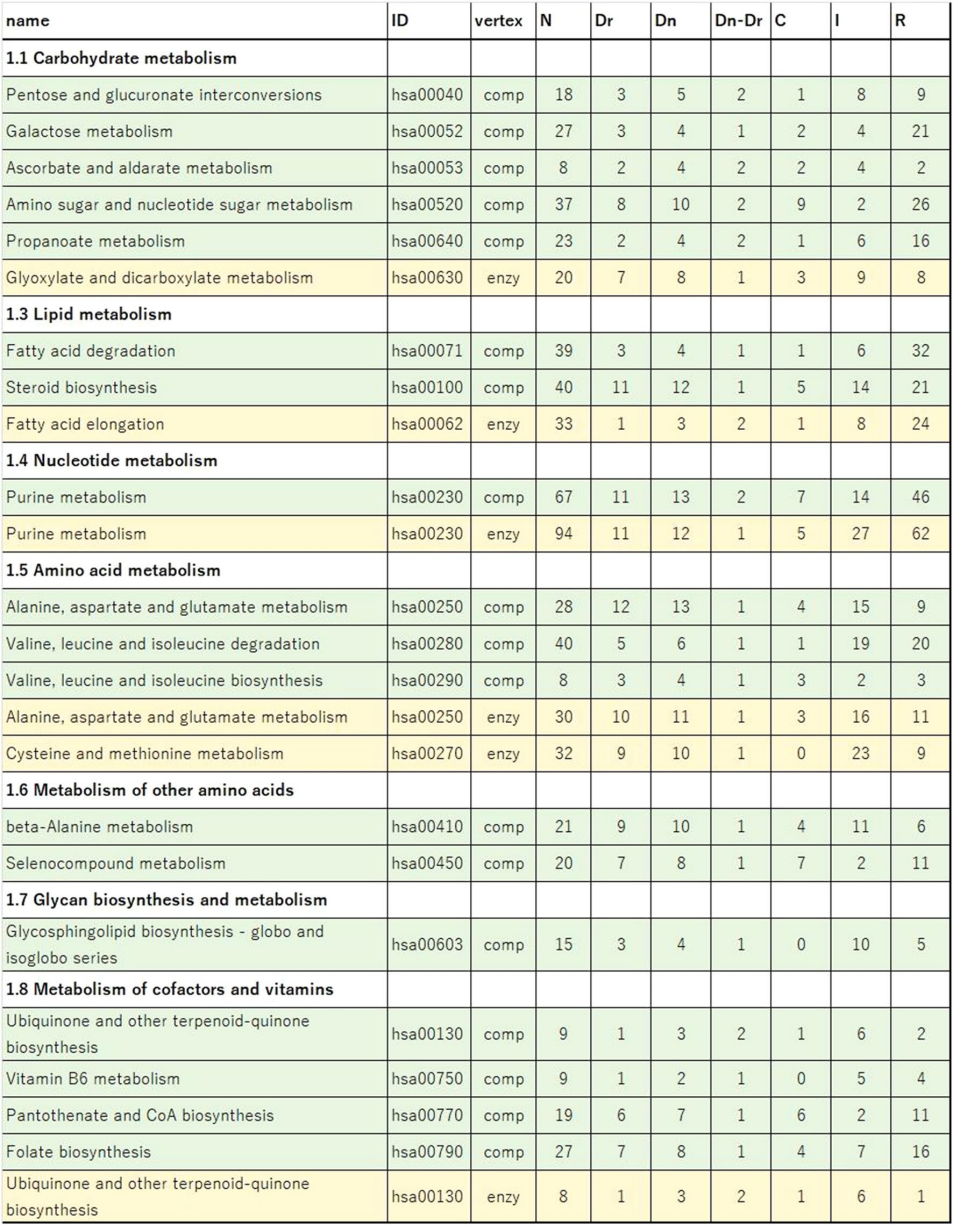
The analysis of human pathways reveals a non-zero difference between the number of driver and driven nodes in 18 chemical compound networks (green) and 6 chemical reaction networks (yellow) (see Supplementary Table S1 online for complete results).

From left to right, the columns indicate: pathway name, KEGG database pathway ID, and the type of network (compound network or chemical reaction (enzymes) network), numbers of nodes N, driver nodes (Dr), driven nodes (Dn) and the observed difference between driven and driver nodes (Dn–Dr). The numbers of identified critical (C), intermittent (I) and redundant (R) nodes are also shown.

For the chemical compound networks, the total difference between driven and driver nodes is 24 chemical compounds. These compounds are multi-signal driven nodes, that is, they are driven by *d*_*m*_ nodes (drivers that send more than one outgoing signal) (see the column driven*-*driver in Table 1). These chemical compounds participate in many other functional pathways in human metabolism (see Fig. 2c and Supplementary Table S2 online), especially in biosynthesis of secondary metabolites and carbon metabolism, including disease pathways such as central carbon metabolism in cancer. For shared functional pathways details of driven chemical reactions see Supplementary Table S3 online. The analysis identified five major human pathways, namely carbohydrates, lipids, nucleotide, amino-acid, and co-factor and vitamin pathways, in which 8 driven chemical reactions are controlled by drivers that send more than one outgoing signal (see Table 1).

We then performed a gene ontology analysis on some of the identified driven genes (see Supplementary Table S4 online) that encode enzymes in these chemical reactions. Genes NQO1 and ACO1 (reactions R02964 and R01900) belong to major cofactors and vitamins metabolism and carbohydrate metabolism, respectively, and are associated with several important biological processes. ACO1 is responsible for intestinal absorption, post-embryonic development and regulation of translation functions. ACO1 is expressed and located in Golgi apparatus and cytoplasm. RNA binding are among the shared molecular functions. Next, NQO1 is responsible for several biological functions from aging to multiple responses to oxidative stress, electrical stimulus, estradiol, and ethanol among others. It is expressed at the cytosol and cytoplasm as well as in the neuronal cell body. Main molecular functions are RNA binding among others (see Supplementary Table S4 online for details on the biological functions of all identified driven genes).

The fact that the identified driven chemical compounds and reactions, that are controlled by multi signal driver nodes, are largely shared by many key pathways highlights the importance of developing controllability methods that do not provide a different number of driver and driven nodes. As shown above, chemical compounds and reactions controlled by drivers that send more than one outgoing signals play relevant functions in the human metabolism, and therefore they should be controlled specifically by driver nodes with single-outgoing signals.

### Identification of critical driven nodes in human metabolic pathways

As stated above, the identification of driven nodes is not unique and it may consist of multiple solutions. Therefore, it is appropriate to define driven control categories such as critical, intermittent and redundant in a similar way that it was established for driver nodes in previous research^1–21^. They are defined as follows:

A critical driven node appears in all minimum driven node sets. An intermittent driven node appears in some but not all minimum driven node sets. A redundant node does not appear in any minimum driven node set.

To identify these unique driven categories, we proposed a novel algorithm using ILP techniques (see “Methods” section for details).

**Figure 3:**
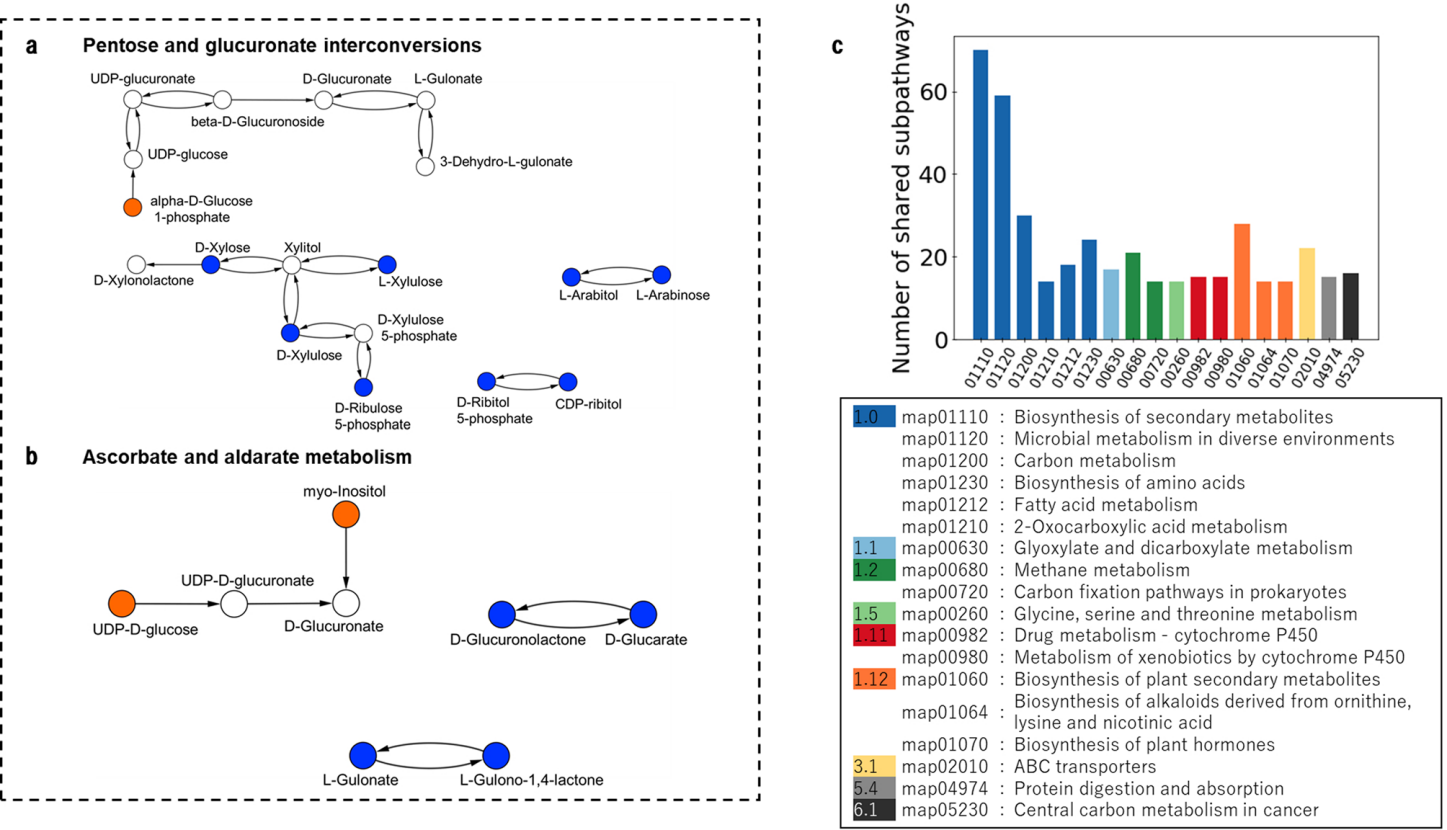
Networks (**a**, **b**) show that same pathways as shown in Fig. 2a,b with nodes classified according to driven control categories using the proposed algorithm. (**a**) While the pentose and glucoronate interconversion metabolism reveals one critical node (orange), (**b**) the ascorbate and aldarate metabolism shows two critical nodes. Blue circles denote intermittent driven nodes and white circles indicates redundant driven nodes. (**c**) The identified critical driven chemical compounds (265) are shared by many other functional pathways in the human metabolism.

Figure 3a,b show that same pathways as shown in Fig. 2a,b with nodes classified according to driven control categories. While the pentose and glucoronate interconversion metabolism (Fig. 3a) reveals one critical driven node (orange), the ascorbate and aldarate metabolism (Fig. 3b) shows two critical driven nodes (orange). Blue denotes intermittent driven nodes and white indicates redundant driven nodes. In both pathways, approximately the 50% of nodes are classified as intermittent driven nodes.

The identified critical chemical compounds (265) participate in many distinct functional pathways in human metabolism (see Fig. 3c). Moreover, some pathways shared by driven chemical compounds that are controlled by multi-signal drivers (*d*_*m*_) are also shared by the critical driven set. Interestingly, biosynthesis of secondary metabolites and carbon metabolism, including carcinogenesis pathways, together with microbial metabolism in diverse environments are among the pathways largely shared by the identified critical driven chemical compounds. (see Figs. 2c and 3c).

The complete analysis of control categories in all human metabolome reveals that most pathways contain several critical driven nodes (see Tables 1 and S6 for the full list). For example, amino sugar and nucleotide sugar metabolism is a key pathway inside the global carbohydrate metabolism. This pathway contains 37 chemical compounds, and among them, 9 compounds are critical driven nodes. The numbers of intermittent and redundant driven nodes identified by our proposed algorithm are also indicated in Tables 1 and S6.

### Analysis of driven nodes in plant metabolic networks

**Figure 4:**
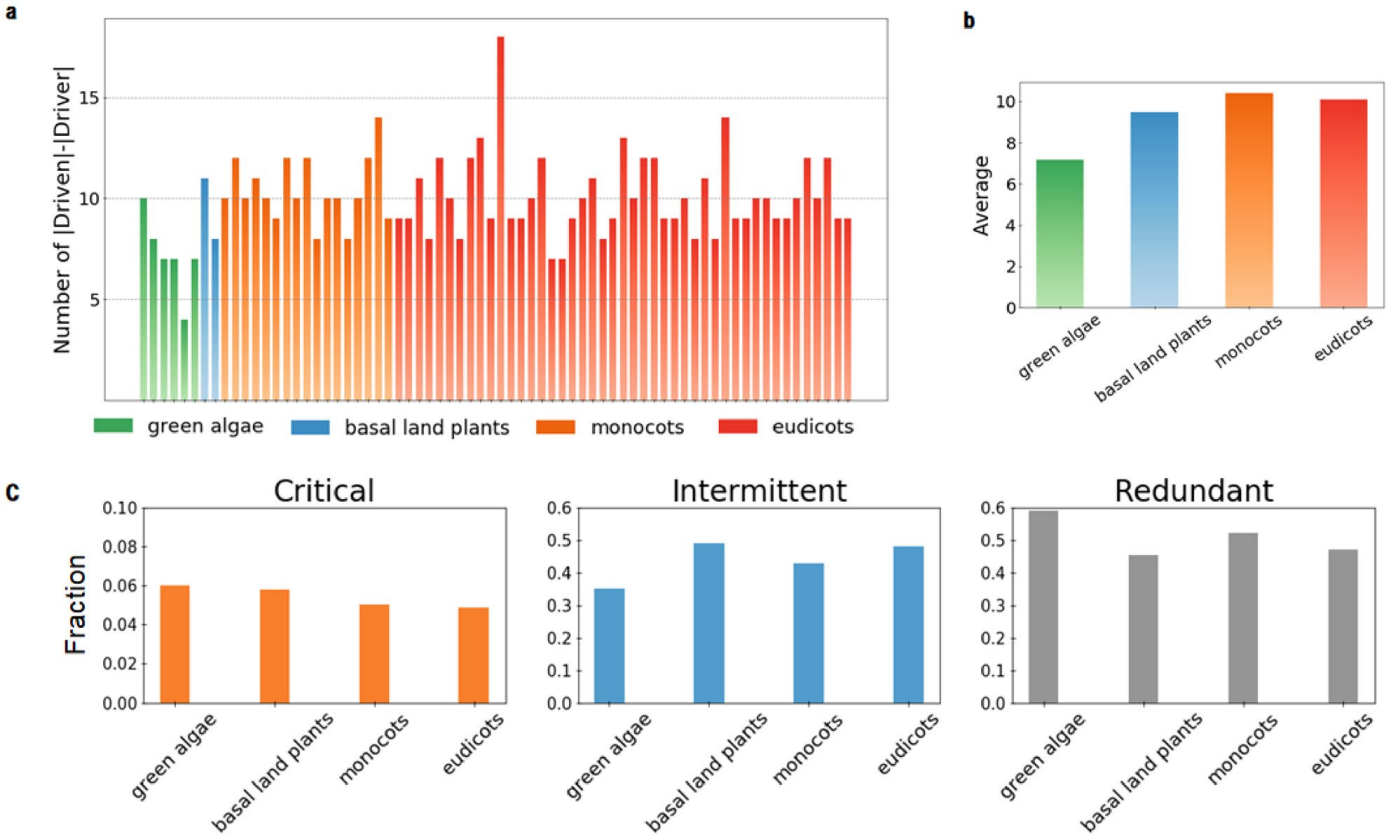
(**a**) The histogram for each plant species of the difference between number of |driven|–|driver| nodes in each plant lineage and (**b**) its average per plant lineage. The average value tends to increase with evolutionary time. (**c**) The fraction of critical, intermittent and redundant driven nodes for each major plant lineage.

From the Plant Metabolic Network Database^27,28^, we collected 70 plant metabolic networks classified into four major groups in the plant lineage: six green algae, two early land plants and angiosperms subdivided into seventeen monocots and forty-four eudicots. The plant pathway analysis was performed using an enzyme/reaction-centric network. First, the results show a systematic difference between the number of driver and driven nodes in all examined networks, without exception. The difference oscillates from 4 to 18 (see Table S5). A histogram for each plant lineage is also shown in Fig. 4a. We then computed the average of the difference in the number of driven and driver nodes in each plant lineage (Fig. 4b). The average value tends to increase with evolutionary time, from the fundamental green algae and early basal plant lineages to more complex and modern angiosperms, including monocots and eudicots, although the tendency is small. This result implicitly suggests an increase in the number of loops and cycles during lineage evolution.

### Identification of critical driven nodes in plant metabolic networks

By using the developed critical driven algorithm, we performed a statistical analysis on the critical, intermittent, and redundant driven enzyme sets controlled by multi-signal driver (*d*_*m*_) nodes. The fraction of critical, intermittent and redundant driven nodes for each major plant lineages are shown in Fig. 4c. The results show that the critical fraction is much smaller (less than 6%) than those of redundant and intermittent nodes. In contrast, the fraction of redundant nodes is the larger one. From an evolutionary viewpoint, the number of critical driven nodes tends to slightly decrease from green algae and basal plants to modern angiosperms.

**Figure 5:**
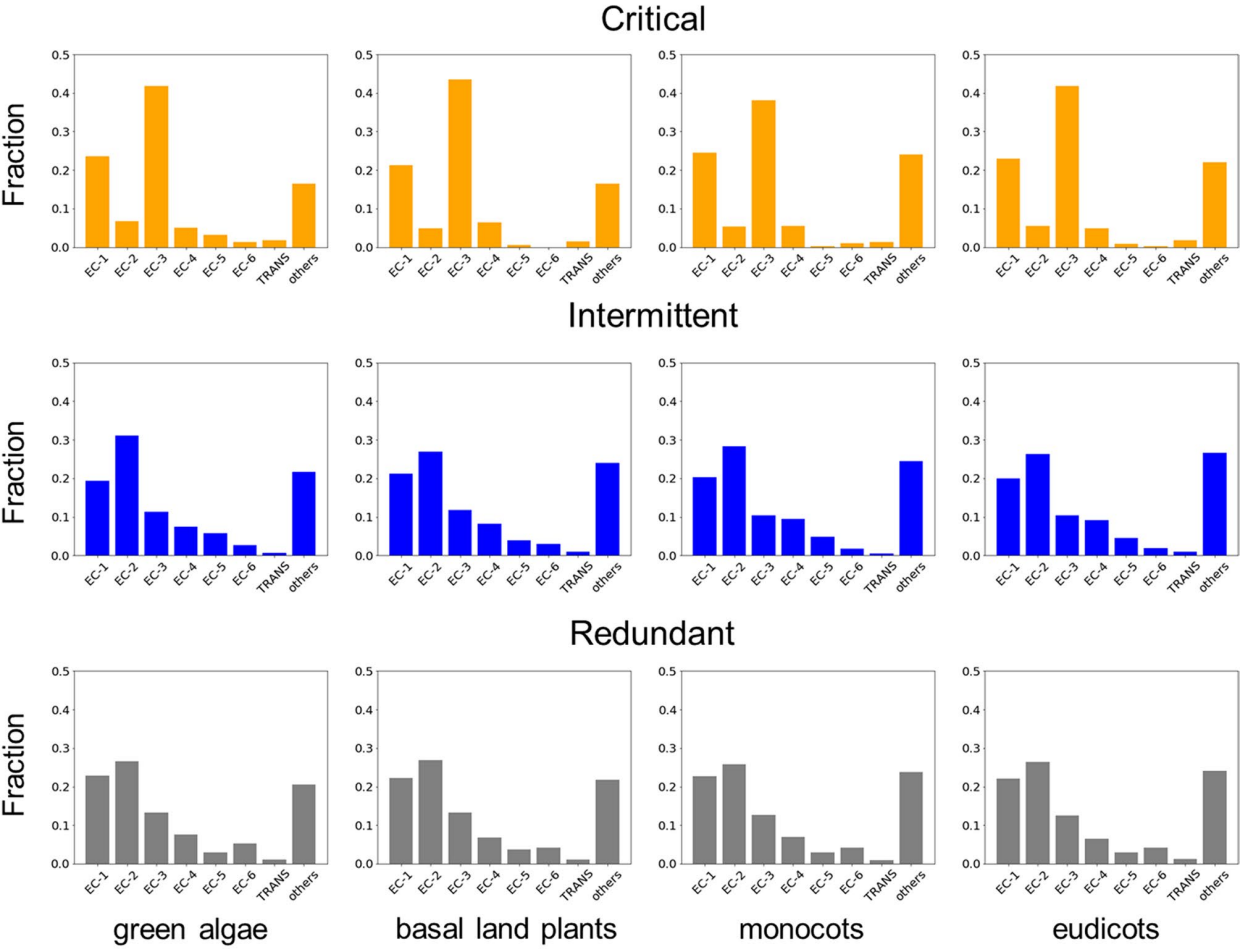
The distribution of critical, intermittent and enzymes according to each EC class and normalized for each plant lineage. Each fraction captures which EC class is more abundant for each plant lineage and control category.

**Figure 6:**
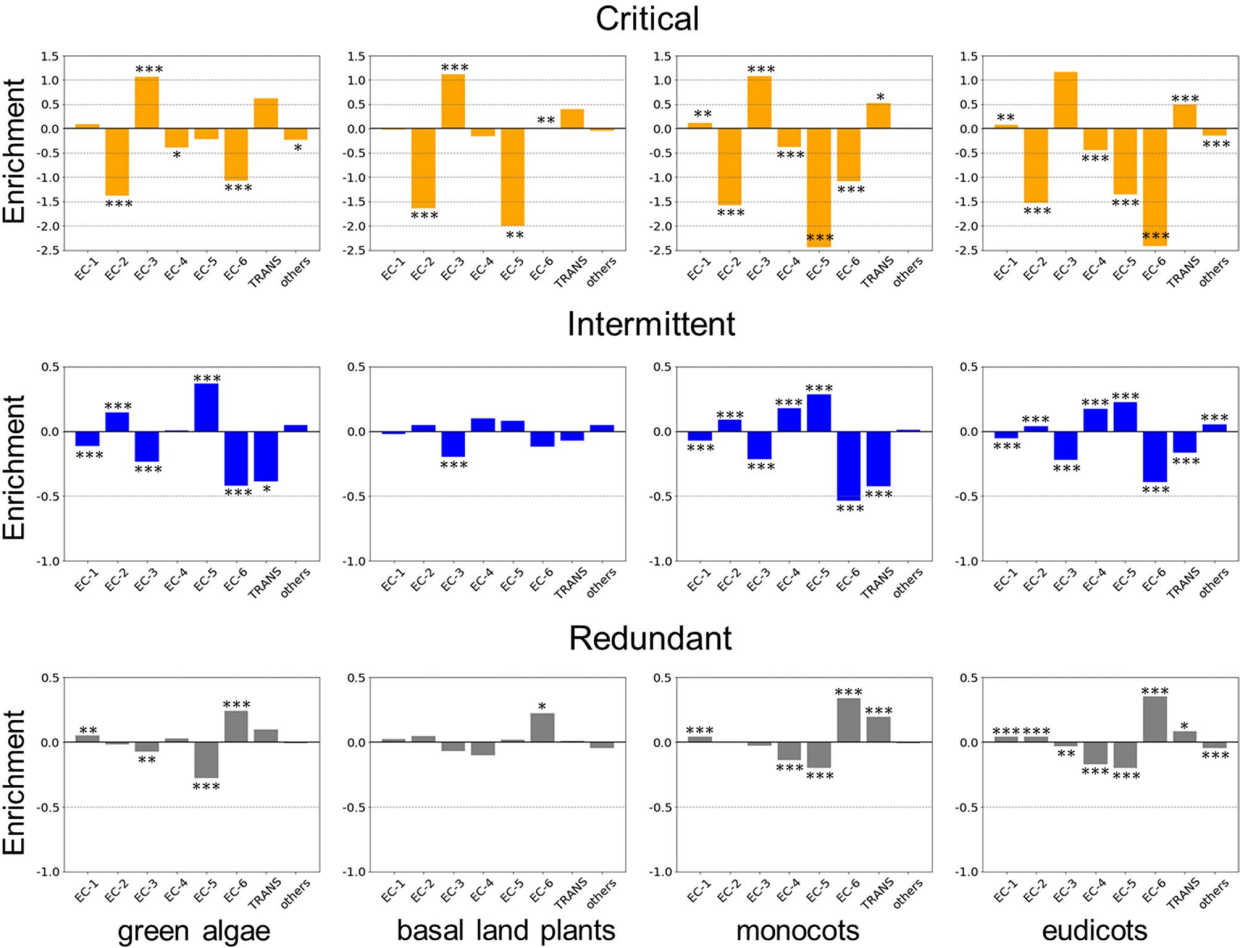
Enrichment or depletion of driven enzymes associated with each control category and classified into EC classes. The fraction of enzymes in each control category and EC class is normalized for each control category. This fraction captures the abundance of enzymes for each EC class in each control category. The results are shown for each plant lineage. The statistical significance of the enrichment or depletion of the identified critical, intermittent and redundant driven enzymes in each EC class is evaluated using fisher’s exact test. The two-tailed exact p-values are denoted in each bar using the following notation: *$p \le 0.05$, **$p \le 0.01$, ***$p \le 0.001$. A detailed list of the exact p-values is shown in Table S7.

The Enzyme Commission (EC) number specifies the type of enzyme-catalysed reactions and can be classified into seven major functional groups^29^. The functional distribution of all control critical enzyme set shows, in general, similar plant lineage-related abundances of critical, intermittent, redundant driven nodes across evolutionary lineages (see Figs. 5 and 6).

Figure 5 shows the fraction $R_{E}^{C} = \sum\limits_{i} {N_{E,i}^{C} /\sum\limits_{i} {N_{i}^{C} } }$ which is defined as the number of nodes in each control category *C* (critical, intermittent and redundant) and in each EC functional group *E* computed for each species *i* $N_{E,i}^{C}$ divided by the number of nodes in each control category *C* for each species *i* $N_{i}^{C}$ and computed for all species *i* in each plant lineages. This metric captures which EC class is more abundant for each plant lineage and control category. For example, in a complete plant linage, EC-3 (hydrolases) and EC-1 (oxidoreductases) enzymes tend to be more engaged in critical driven control. Moreover, this pattern is conserved across lineages. In contrast, EC-2 (transferases) enzyme class tends to be more dominant among those enzymes engaged in intermittent and redundant roles.

Supplementary Fig. S3 online shows the fraction $F_{E}^{C} = \sum\limits_{i} {F_{E,i}^{C} } /L$ where $F_{E,i}^{C} = N_{E}^{C} /N_{E,i}$ . This metric is defined as the number of nodes in each control category C (critical, intermittent, and redundant enzymes) and in each EC functional group *E,* $N_{E}^{C}$, and divided by the number of nodes in each EC functional a group and species *i*, $N_{E,i}$, and averaged for all species (*L*) in each plant lineage ($F_{E}^{C}$). Differently from Fig. 5, this metric captures the relative abundance of enzymes in each control category for each EC class. Therefore, the sum of the fractions for the three control categories gives 1 for each EC class. Among all EC classes, and in fair agreement with Fig. 5, EC-3 class tends to have the largest fraction of critical driven enzymes. It is remarkable that some EC-classes such as EC-5 (isomerases) and EC-6 (ligases) are composed of enzymes with lowest participation in critical roles.

### Enrichment analysis

We performed an enrichment analysis on the EC-classes for each plant lineage. We first evaluated the fraction of enzymes identified for a given driven control category (T) (critical, intermittent and redundant) and present in a given EC functional class (P) $N_{T}^{P}$ divided by the total number of enzymes identified for the given category (T) $N_{T}^{{}}$, which leads to $f_{T}^{P} = N_{T}^{P} /N_{T}$. We then computed the fraction of enzymes that belong to a given enzyme class $N^{P}$ divided by the total number of nodes *N* in a given plant metabolic network $f_{{}}^{P} = N_{{}}^{P} /N_{{}}$. Then the enrichment factor for a control category T in a given functional class (P) is given by $E_{T}^{P} = \ln (f_{T}^{P} /f^{P} ).$

Next, the statistical significance of the enrichment or depletion of the identified critical, intermittent and redundant driven enzymes in each EC class was evaluated using Fisher’s exact test. The two-tailed exact p-values are denoted in each bar using the following notation: *$p \le 0.05$, **$p \le 0.01$,***$p \le 0.001$. The results are shown in Fig. 6. In agreement with results shown in Fig. 5 and Supplementary Fig. S3, the EC-3 (hydrolases) class tends to have the largest statistical enrichment ($p \le 0.001$) for critical driven enzymes. This enrichment is also conserved across evolutionary lineages. In contrast, EC-2 in all lineages and EC-5 and EC-6 classes for monocots and eudicots groups show the largest depletion of critical driven enzymes ($p \le 0.001$). However, EC-5 (isomerases) class shows a positive enrichment of intermittent driven enzymes across lineages so the enzymes are actively engaged in control roles. Interestingly, it also shows the largest depletion of redundant driven enzymes ($p \le 0.001$) except for basal land plants.

**Figure 7:**
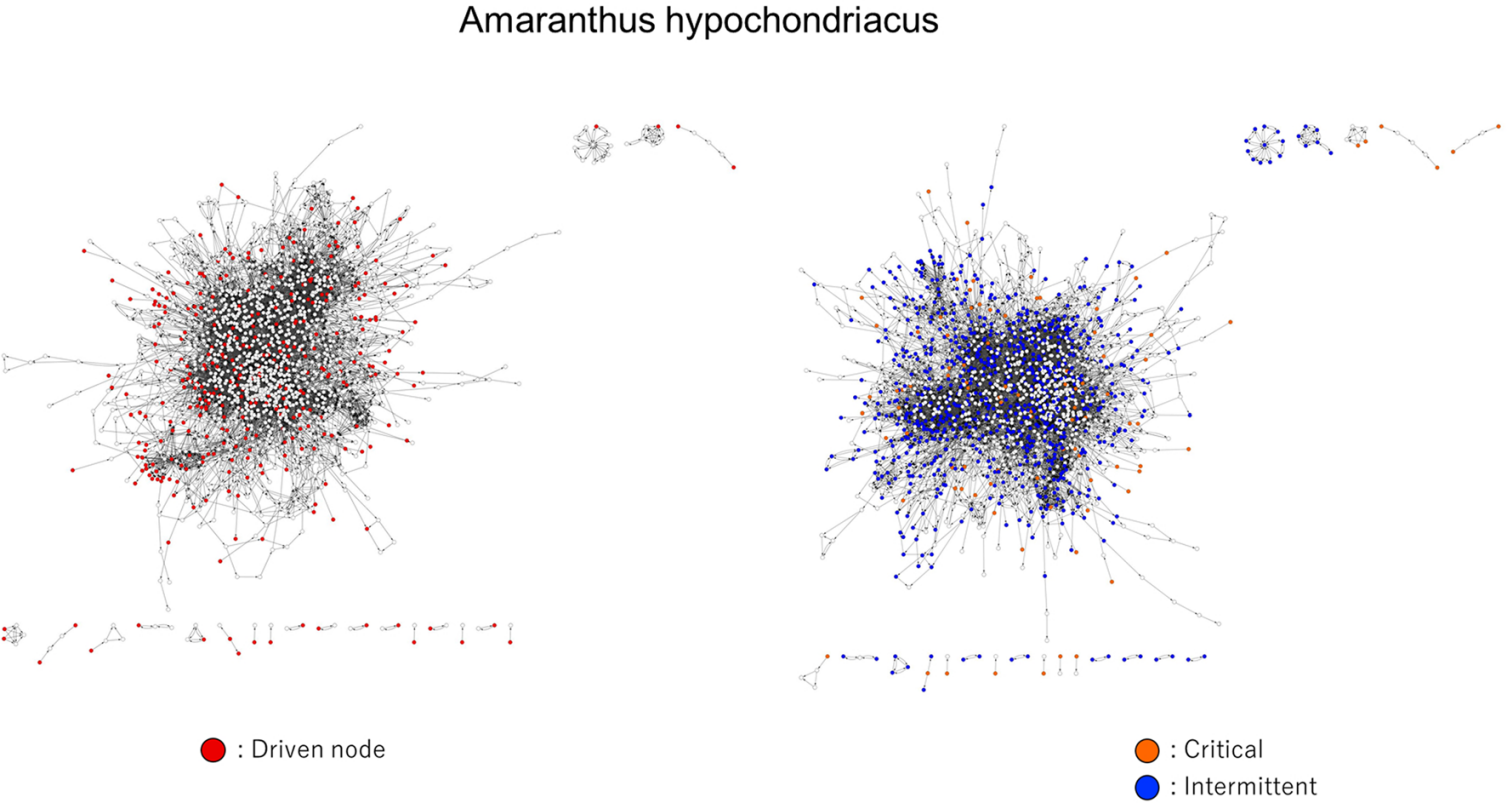
Network visualization of the *Aramanthus hypochondriacus* plant, commonly known as prince’s feather, that belongs to the eudicot plant lineage. Left: Identified driven enzymes are denoted in red. Right: Identified critical, intermittent, and redundant driven enzymes are denoted in orange, blue and white, respectively.

Although a large fraction of the driven reactions, controlled by driver nodes that send more than one outgoing signals, were identified in isolated components (see Fig. 7), we also identified enzymes located in the main component controlled by multi-signal driver nodes. This finding suggests that additional driven nodes are required to control subcomponents, which is also ignored in the maximum matching-based approach.

## Discussion

The MM method has been widely applied to cell biology and technological systems. However, the MM assumes that one driver node can send control signal to many other nodes. This assumption may not be practical in certain kinds of complex systems, especially in the control of biological networks. Indeed, our extensive data-driven analysis of metabolic pathways demonstrates that the MM approach leads to a number of driver nodes that is smaller than that of the driven nodes. As we have discussed, this difference increases with the number of cycles and loops in the network. Moreover, those driven nodes controlled by multi-signal driver nodes, are associated to important biological functions or are largely shared by other functional pathways. Therefore, controllability models that add constraints to the MM to cope with the difference of the number of driver and driven nodes are necessary. This is the first work that shows the importance of use of driven nodes in analysis of real-world networks.

On the other hand, we have presented a novel algorithm that uses the concept of control categories to determine uniquely driven nodes. We then applied this algorithm to determine critical driven nodes in metabolic pathways. The analysis was used to assess the biological functions of specific critical driven nodes in metabolic pathways. To our best knowledge, this is the first algorithm that enables us to efficiently identify critical driven nodes in large real-world networks.

Clearly, the method based on MM works very well in many real systems, in particular, as discussed above, in those in which the number of loops or cycles is small. Therefore, this work does not aim to criticize the maximum matching-based approach but to complement it. We hope that this work stimulates further studies on the structural controllability analysis of complex networks in more practical and feasible settings.

## Methods

### Driven nodes algorithm

Our study is based on the work by Pequito et al.^19^ to identify the minimum set of driven nodes. Here we review their algorithm, which is referred to as *IdentifyDriven*(*G*(*V,E*)) where *G*(*V,E)* is a given directed network and *V* = {*v*_1_, …,* v*_*n*_}*.* The purpose of the algorithm is to output a minimum set of driven nodes *V*_*D*_ for a given *G*(*V,E*).

1. Let *N*_*i*_ (*i* ∈ {1*, . . . ,*$\beta$}) be the set of nodes in the *i*th non-top linked SCC (strongly connected component), that is, an SCC without an incoming edge from another SCC (including the case of single node).
2. Construct a set of new nodes $U = \{ 1, \ldots ,u_{\beta } \}$, which is to be used in a bipartite graph constructed in STEP (3).
3. Construct a bipartite graph *B*(*V*_*L*_*, V*_*R*_*, E*_*B*_) by
  $$V_{L} = \{ x_{1} , \ldots , x_{n} \} \cup U,$$
  $$V_{R} = \{ y_{1}, \ldots , y_{n} \} ,$$
  $$E_{{\text{B}}} = \{ \left( {x_{i} , \, y_{j} } \right)|\left( {v_{i} , \, v_{j} } \right) \in E\} \cup \{ \left( {u_{i} , \, y_{j} } \right)|u_{i} \in U,v_{j} \in N_{i} \} .$$
4. Give weight − 1 (resp., weight − 2) to each edge (*x*_*i*_*, y*_*j*_) (resp., (*u*_*i*_*, y*_*j*_)).
5. Compute a maximum matching *M* with the minimum weight (the total weight of matched edges is minimum under the condition of the maximum number of edges).
6. Let *D*_*R*_ = {*y*_*i*1_*,…,y*_*ik*_} (⊆ *V*_*R*_) be the set of nodes matched with *u*_*i*_s and non-matched nodes in *V*_*R*_ with respect to *M* (see Fig. 1g–j).
7. Return *V*_*D*_ = {*v*_*ij*_ | *v*_*ij*_ ∈ *D*_*R*_ } as a minimum set of driven nodes.

The necessity of *u*_*i*_s is illustrated in Supplementary Fig. S1 online, which shows that it is difficult to determine the driven node(s) if we do not use *u*_*i*_s.

The minimum weight maximum bipartite matching can be computed in polynomial time using Hungarian algorithm^19^. However, for ease of implementation, we describe an ILP based method in the Supplementary Information file.

### Novel algorithm for identification of critical driven nodes

Here, we present our novel algorithm to determine critical/intermittent driven nodes and the remaining redundant nodes. We define critical/intermittent driven nodes and redundant nodes as below.

*Critical node*: appearing in all minimum driven node sets.

*Intermittent node*: appearing in some but not all minimum driven node sets.

*Redundant*: not appearing in any minimum driven node set.

**Figure 8:**
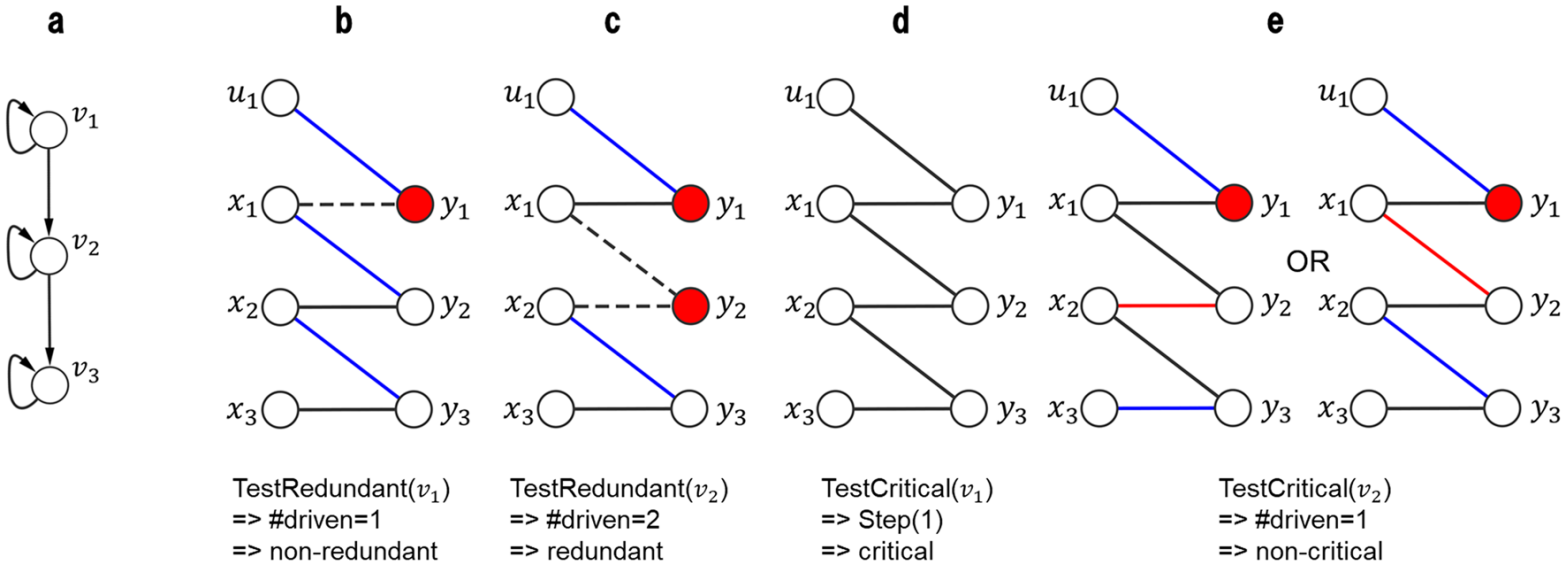
Illustration of the novel algorithm for critical/redundant driven nodes identification. (**a**) Original network. (**b**, **c**) Example of TestRedundant(*v*_*i*_) procedure. Dashed edges are those deleted at Step (2) of TestRedundant(*v*_*i*_). (**d**, **e**) Example of TestCritical(*v*_*i*_) procedure. Red edges are those that are forced to put in *M* at Step (4) of TestCritical(*v*_*i*_). Red nodes indicate driven nodes. Blue edges denote matching link.

The following is a procedure (*TestRedundant*(*v*_*i*_)) to decide whether or not a given node *v*_*i*_ is a redundant driven node for a given graph *G*(*V,E*).

(see Fig. 8 and Supplementary Fig. S2 online).

1. Construct (*V*_*L*_*, V*_*R*_*, E*_*B*_) as in STEP (1)-(4) of *IdentifyDriven*(*G*(*V,E*)).
2. Delete all edges connected to *y*_*i*_*.*
3. If *y*_*i*_ is in a non-top linked SCC, delete edges from *u*_*j*_ where *u*_*j*_ corresponds to the SCC. (This operation implicitly means that (*u*_*j*_*, y*_*i*_) is added to *M*.)
4. Compute a maximum matching *M* with the minimum weight, and then determine the driven nodes as in Step (6)-(7) of *IdentifyDriven*(*G*(*V,E*)). If the number of driven nodes increases, return TRUE. Otherwise, return FALSE.

Note that Step (2) puts a constraint that *v*_*i*_ must be the first node in a stem.

The following is a procedure (*TestCritical*(*v*_*i*_)) to decide whether or not a given node *v*_*i*_ is a critical driven node for a given graph *G*(*V,E*).

1. If there is no *v*_*j*_ such that (*v*_*j*_, *v*_*i*_) ∈ *E* with $v_{j} \ne v_{i}$, *v*_*i*_ is critical. Otherwise, do Steps (2)–(7).
2. Construct (*V*_*L*_*, V*_*R*_*, E*_*B*_) as in STEP (1)–(4) of *IdentifyDriven*(*G*(*V,E*)).
3. For all *v*_*j*_ ∈ *V* such that (*v*_*j*_*, v*_*i*_) ∈ *E*, do Steps (4)–(6)
4. Compute a maximum matching *M* with the minimum weight under the condition that (*x*_*j*_*, y*_*i*_) appears in *M*.
5. Determine the driven nodes from *M* as in STEP (6)–(7) of *IdentifyDriven*(*G*(*V,E*)).
6. If the number of driven nodes does not increase, return FALSE.
7. Return TRUE.

Note that Step (4) puts a constraint that *v*_*i*_ cannot be the first node in a stem. If we use Hungarian algorithm, we can implement Steps (3)–(7) by applying it repeatedly for each *x*_*j*_ connected to *y*_*i*_ with deleting the edges connected to *x*_*j*_ and the edges connected to *y*_*i*_. If we use ILP, we can implement Steps (3)–(7) by adding the following constraint on *v*_*i*_:

$$\sum\limits_{{(v_{j} ,v_{i} ) \in E}} {z_{j,i} } = 1$$

Note also that the intermittent driven nodes are obtained by removing the critical driven nodes and the redundant nodes from *V*.

## Supplementary Information

Supplementary Information 1.

Supplementary Table S1.

Supplementary Table S2.

Supplementary Table S3.

Supplementary Table S4.

Supplementary Table S5.

Supplementary Table S6.

Supplementary Table S7.

## References

[1] Liu Y-Y, Slotine J-J, Barabási A-L (2011). Controllability of complex networks. Nature.

[2] Nacher JC, Akutsu T (2012). Dominating scale-free networks with variable scaling exponent: Heterogeneous networks are not difficult to control. New J. Phys..

[3] Nepusz T, Vicsek T (2012). Controlling edge dynamics in complex networks. Nat. Phys..

[4] Mochizuki A, Fiedler B, Kurosawa G, Saito D (2013). Dynamics and control at feedback vertex sets. II: A faithful monitor to determine the diversity of molecular activities in regulatory networks. J. Theor. Biol..

[5] Yuan Z, Zhao C, Di Z, Wang W-X, Lai Y-C (2013). Exact controllability of complex networks. Nat. Commun..

[6] Wuchty S (2014). Controllability in protein interaction networks. Proc. Natl. Acad. Sci. USA.

[7] Gao J, Liu Y-Y, D'Souza RM, Barabási A-L (2014). Target control of complex networks. Nat. Commun..

[8] Vinayagam A (2016). Controllability analysis of the directed human protein interaction network identifies disease genes and drug targets. Proc. Natl. Acad. Sci. USA.

[9] Basler G, Nikloaki Z, Larhlimi A, Barabási A-L, Liu Y-Y (2016). Control of fluxes in metabolic networks. Genome. Res..

[10] Zañudo JGT, Yang G, Albert R (2017). Structure-based control of complex networks with nonlinear dynamics. Proc. Natl. Acad. Sci. USA.

[11] Yan G (2017). Network control principles predict neuron function in the *Caenorhabditis elegans* connectome. Nature.

[12] Kim JZ (2018). Role of graph architecture in controlling dynamical networks with applications to neural systems. Nat. Phys..

[13] Hu Y (2019). Optimal control nodes in disease-perturbed networks as targets for combination therapy. Nat. Commun..

[14] Schwartz J-M, Otokuni H, Akutsu T, Nacher JC (2019). Probabilistic controllability approach to metabolic fluxes in normal and cancer tissues. Nat. Commun..

[15] Lin CT (1974). Structural controllability. IEEE Trans. Autom. Control.

[16] Cowan NJ, Chastain EJ, Vilhena DA, Freudenberg JS, Bergstrom CT (2012). Nodal dynamics, not degree distributions, determine the structural controllability of complex networks. PLoS ONE.

[17] Ruths J, Ruths D (2014). Control profiles of complex networks. Science.

[18] Campbell C, Ruths J, Ruths D, Shea K, Albert R (2016). Topological constraints on network control profiles. Sci. Rep..

[19] Pequito S, Kar S, Aguiar AP (2016). A framework for structural input/output and control configuration selection in large-scale systems. IEEE Trans. Autom. Control.

[20] Jia T (2013). Emergence of bimodality in controlling complex networks. Nat. Commun..

[21] Nacher JC, Akutsu T (2014). Analysis of critical and redundant nodes in controlling directed and undirected complex networks using dominating sets. J. Complex Netw..

[22] Campbell C, Aucott S, Ruths J, Ruths D, Shea K, Albert R (2017). Correlations in the degeneracy of structurally controllable topologies for networks. Sci. Rep..

[23] Liu Y-Y (2016). Control principles of complex systems. Rev. Mod. Phys..

[24] Czeizler E, Wu C-K, Gratie C, Kanhaiya K, Petre I (2018). Structural target controllability of linear networks. IEEE/ACM Trans. Comput. Biol. Bioinf..

[25] Czeizler E, Popa A, Popescu V, Jansson J, Martín-Vide C, Vega-Rodríguez M (2018). Fixed parameter algorithms and hardness of approximation results for the structural target controllability problem. Algorithms for Computational Biology. AlCoB 2018. Lecture Notes in Computer Science.

[26] Kanehisa M, Sato Y, Furumichi M, Morishima K, Tanabe M (2019). New approach for understanding genome variations in KEGG. Nucl. Acids Res..

[27] Chae L, Kim T, Nico-Poyanco R, Rhee SY (2014). Genomic signatures of specialized metabolism in plants. Science.

[28] Rhee, S. Y. *Plant Metabolic Network Database Version 16.0 (PMN)*. www.plantcyc.org.

[29] Enzyme Database. *ExPASy Bioinformatics Resource Portal:* https://enzyme.expasy.org/. Accessed 31 Jul 2019.

