# Supplementary material for: Uncovering and classifying the role of driven nodes in control of complex networks
Source: Sci Rep. 2021 May 5;11:9627. doi: 10.1038/s41598-021-88295-4 (PMC8100151; doi:10.1038/s41598-021-88295-4)
Supplement: Supplementary file 1 — Supplementary Information 1. [file 41598_2021_88295_MOESM1_ESM.pdf]

Supplementary Information

Uncovering and classifying the role of driven nodes in control of complex networks

Yuma Shinzawa, Tatsuya Akutsu & Jose C. Nacher

I. ILP for minimum weight maximum matching

II. Supplementary Information Figures S1-S3

III. Supplementary Information Tables: Tables S1-S7.

Excel Tables S1-S5, S7 and PDF Table S6 can be downloaded electronically.

ILP for minimum weight maximum matching

Let $w_{i,j}$ be a weight for an edge (v_i, v_j), where $w_{i,j} = -1$ or $w_{i,j} = -2$. Let D be an integer such that $D \gg n$. Let E' denote the set of edges between $V_L \cup U$ and V_R.

maximize $$\sum_{(v_i,v_j)\in E'} (-w_{i,j} + D) z_{i,j} \qquad (1)$$

subject to $$\sum_{(v_j,v_i)\in E'} z_{j,i} \le 1 \qquad \text{for all } v_j \in V_L \cup U, \qquad (2)$$

$$\sum_{(v_j,v_i)\in E'} z_{j,i} \le 1 \qquad \text{for all } v_i \in V_R\ , \qquad (3)$$

$$z_{i,j} \quad \in \{0, 1\} \qquad \text{for all } (v_i, v_j) \in E'. \qquad (4)$$

Supplementary Information Figures

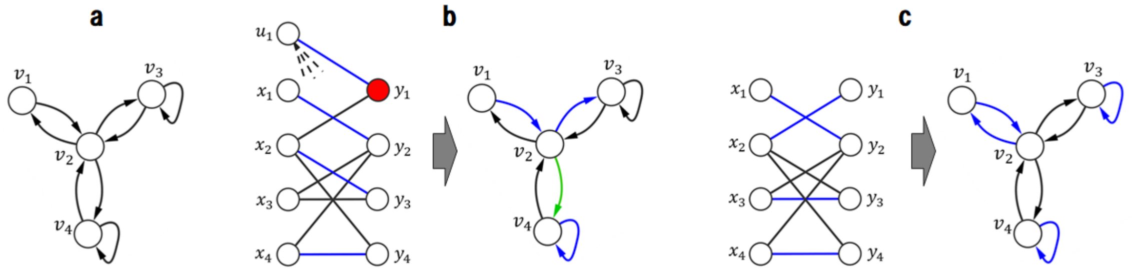

Fig. S1 (**a**) Original network *G(V, E)*. (**b**) Bipartite graph with u_is, and the corresponding matching (blue) and subgraph (green+blue). (**c**) Bipartite graph without u_is and the corresponding matching (blue) and subgraph (blue).

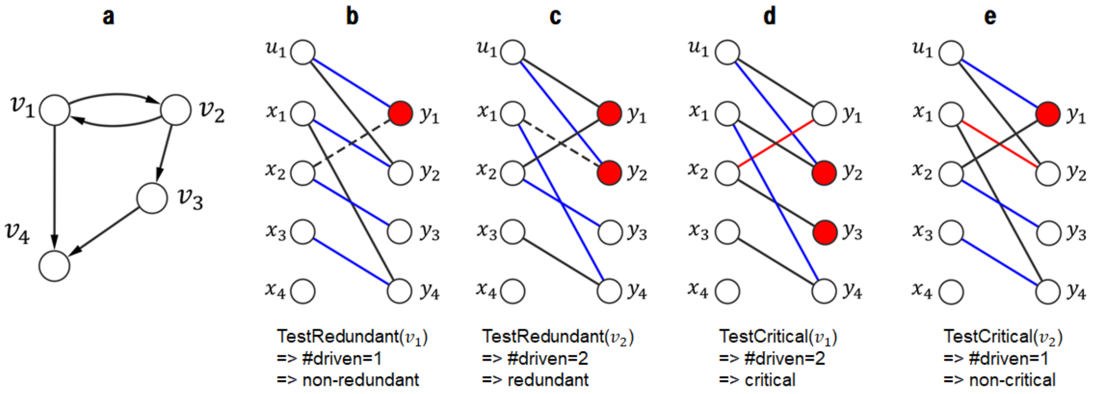

Fig. S2 Additional examples of TestRedundant(v_i)/TestCritical(v_i).

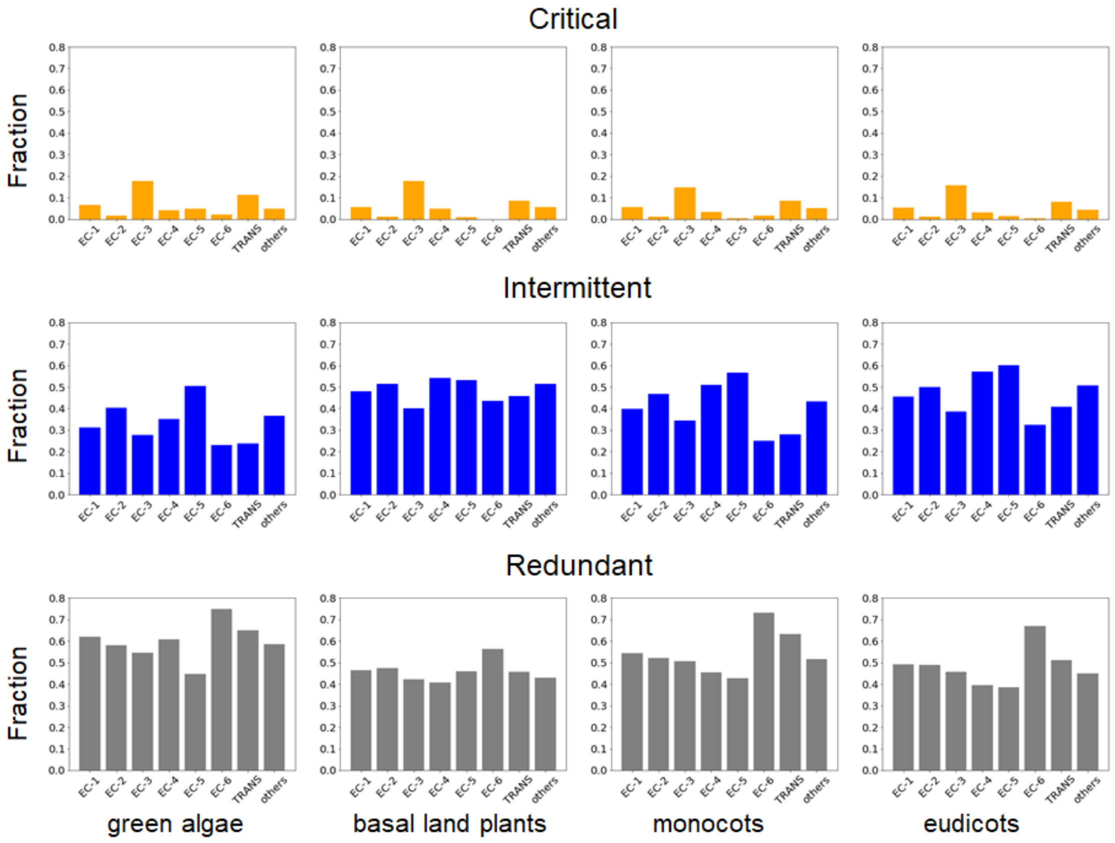

Fig. S3 The fraction of enzymes in each control category and EC class normalized for each control category. This fraction captures the abundance of enzymes for each EC class in each control category. The results are shown for each plant lineage.

Table S1. (Excel file) The analysis of human pathways reveals a non-zero difference between the number of driver and driven nodes in 18 chemical compound networks (green) and 6 chemical reaction networks (yellow). This table shows the complete results for all the analyzed pathways. From left to right, the columns indicate: pathway name, KEGG database pathway ID, and the type of network (compound network or chemical reaction (enzymes) network), numbers of nodes *N*, driver nodes, driven nodes and the observed difference between driven and driver nodes.

Table S2. (Excel file) Background data of Fig. 3c. From left to right, the columns indicate Pathway ID, driven node (chemical compound ID), shared pathway ID and pathway name. All IDs refer to KEGG database.

Table S3. (Excel file). Shared pathways of driven enzymes controlled multi-signal driver nodes. From left to right, the columns indicate Pathway ID, driven node (chemical reaction ID), shared pathway ID and pathway name. All IDs refer to KEGG database.

Table S4. (Excel file). Gene Ontology data associated to all identified driven enzymes controlled by multi-signal driver nodes. First sheet indicates the gene symbol of each enzyme. Next sheets show the associate GO terms for each gene from biological process, cellular component, and molecular function, respectively.

Table S5. (Excel file). Background data of Fig. 5.

Table S6. (PDF file). Data for all analyzed human metabolic pathways. From left to right, each column indicates: pathway name, KEGG database pathway ID, type of network (compound network or chemical reaction (enzymes) network) number of nodes *N*, number of driven nodes, and the number of critical, intermittent and redundant driven nodes in each pathway.

Table S7. (Excel file). A detailed list of the exact p-values corresponding to Fig. 7.
