# Supplementary material for: Uncovering and classifying the role of driven nodes in control of complex networks
Source: Sci Rep. 2021 May 5;11:9627. doi: 10.1038/s41598-021-88295-4 (PMC8100151; doi:10.1038/s41598-021-88295-4)
Supplement: Supplementary file 7 — Supplementary Table S6. [file 41598_2021_88295_MOESM7_ESM.pdf]

name	ID	vertex	N	Driven	Critical	Intermittent	Redundant
**1.1 Carbohydrate metabolism**							
Glycolysis / Gluconeogenesis	hsa00010	compound	26	2	1	2	23
Citrate cycle (TCA cycle)	hsa00020	compound	20	1	1	0	19
Pentose phosphate pathway	hsa00030	compound	22	5	2	5	15
Pentose and glucuronate interconversions	hsa00040	compound	18	5	1	8	9
Fructose and mannose metabolism	hsa00051	compound	20	5	4	2	14
Galactose metabolism	hsa00052	compound	27	4	2	4	21
Ascorbate and aldarate metabolism	hsa00053	compound	8	4	2	4	2
Starch and sucrose metabolism	hsa00500	compound	18	7	6	2	10
Amino sugar and nucleotide sugar metabolism	hsa00520	compound	37	10	9	2	26
Inositol phosphate metabolism	hsa00562	compound	30	10	4	11	15
Pyruvate metabolism	hsa00620	compound	22	4	1	6	15
Glyoxylate and dicarboxylate metabolism	hsa00630	compound	32	10	7	7	18
Propanoate metabolism	hsa00640	compound	23	4	1	6	16
Butanoate metabolism	hsa00650	compound	15	3	3	0	12
Glycolysis / Gluconeogenesis	hsa00010	enzyme	33	3	1	8	24
Citrate cycle (TCA cycle)	hsa00020	enzyme	21	1	1	0	20
Pentose phosphate pathway	hsa00030	enzyme	22	1	1	0	21
Pentose and glucuronate interconversions	hsa00040	enzyme	12	2	1	4	7
Fructose and mannose metabolism	hsa00051	enzyme	19	5	4	2	13
Galactose metabolism	hsa00052	enzyme	20	2	2	0	18
Ascorbate and aldarate metabolism	hsa00053	enzyme	2	1	1	0	1
Starch and sucrose metabolism	hsa00500	enzyme	22	9	6	5	11
Amino sugar and nucleotide sugar metabolism	hsa00520	enzyme	37	8	8	0	29
Inositol phosphate metabolism	hsa00562	enzyme	37	8	2	13	22
Pyruvate metabolism	hsa00620	enzyme	23	4	2	4	17
Glyoxylate and dicarboxylate metabolism	hsa00630	enzyme	20	8	3	9	8
Propanoate metabolism	hsa00640	enzyme	22	3	3	0	19
Butanoate metabolism	hsa00650	enzyme	13	2	2	0	11
**1.2 Energy metabolism**							
Nitrogen metabolism	hsa00910	compound	6	3	2	2	2
Sulfur metabolism	hsa00920	compound	7	2	2	0	5
	ko680	compound	81	13	9	12	60
	ko710	compound	22	5	1	11	10
	ko720	compound	44	4	2	4	38
Nitrogen metabolism	hsa00910	enzyme	3	2	1	2	0
Sulfur metabolism	hsa00920	enzyme	7	2	2	0	5
	ko680	enzyme	91	17	5	22	64
	ko710	enzyme	26	4	0	9	17

	ko720	enzyme	55	7	2	17	36
**1.3 Lipid metabolism**							
Fatty acid biosynthesis	hsa00061	compound	40	6	1	21	18
Fatty acid elongation	hsa00062	compound	38	4	4	0	34
Fatty acid degradation	hsa00071	compound	39	4	1	6	32
Synthesis and degradation of ketone bodies	hsa00072	compound	5	1	0	5	0
Steroid biosynthesis	hsa00100	compound	40	12	5	14	21
Primary bile acid biosynthesis	hsa00120	compound	46	10	4	12	30
Steroid hormone biosynthesis	hsa00140	compound	85	33	5	46	34
Glycerolipid metabolism	hsa00561	compound	16	4	3	3	10
Glycerophospholipid metabolism	hsa00564	compound	37	12	6	11	20
Ether lipid metabolism	hsa00565	compound	20	8	5	6	9
Arachidonic acid metabolism	hsa00590	compound	36	19	1	23	12
Linoleic acid metabolism	hsa00591	compound	5	3	1	3	1
alpha-Linolenic acid metabolism	hsa00592	compound	13	5	5	0	8
Sphingolipid metabolism	hsa00600	compound	21	6	4	4	13
Biosynthesis of unsaturated fatty acids	hsa01040	compound	36	18	12	11	13
Fatty acid biosynthesis	hsa00061	enzyme	38	6	0	13	25
Fatty acid elongation	hsa00062	enzyme	33	3	1	8	24
Fatty acid degradation	hsa00071	enzyme	34	3	2	2	30
Synthesis and degradation of ketone bodies	hsa00072	enzyme	5	1	0	5	0
Steroid biosynthesis	hsa00100	enzyme	35	9	6	6	23
Primary bile acid biosynthesis	hsa00120	enzyme	45	9	5	10	30
Steroid hormone biosynthesis	hsa00140	enzyme	97	34	5	57	35
Glycerolipid metabolism	hsa00561	enzyme	16	4	0	10	6
Glycerophospholipid metabolism	hsa00564	enzyme	47	12	3	19	25
Ether lipid metabolism	hsa00565	enzyme	17	7	3	9	5
Arachidonic acid metabolism	hsa00590	enzyme	36	20	1	24	11
Linoleic acid metabolism	hsa00591	enzyme	4	2	1	3	0
alpha-Linolenic acid metabolism	hsa00592	enzyme	7	4	4	0	3
Sphingolipid metabolism	hsa00600	enzyme	30	7	3	7	20
Biosynthesis of unsaturated fatty acids	hsa01040	enzyme	11	5	4	2	5
**1.4 Nucleotide metabolism**							
Purine metabolism	hsa00230	compound	67	13	7	14	46
Pyrimidine metabolism	hsa00240	compound	41	6	3	29	9
Purine metabolism	hsa00230	enzyme	94	12	5	27	62
Pyrimidine metabolism	hsa00240	enzyme	62	11	2	18	42
**1.5 Amino acid metabolism**							
Arginine biosynthesis	hsa00220	compound	14	4	3	2	9
Alanine, aspartate and glutamate metabolism	hsa00250	compound	28	13	4	15	9

Glycine, serine and threonine metabolism	hsa00260	compound	33	11	4	13	16
Cysteine and methionine metabolism	hsa00270	compound	30	7	2	10	18
Valine, leucine and isoleucine degradation	hsa00280	compound	40	6	1	19	20
Valine, leucine and isoleucine biosynthesis	hsa00290	compound	8	4	3	2	3
Lysine degradation	hsa00310	compound	26	7	6	2	18
Arginine and proline metabolism	hsa00330	compound	38	12	6	11	21
Histidine metabolism	hsa00340	compound	16	5	3	4	9
Tyrosine metabolism	hsa00350	compound	42	13	4	17	21
Phenylalanine metabolism	hsa00360	compound	10	4	1	6	3
Tryptophan metabolism	hsa00380	compound	40	16	3	22	15
Phenylalanine, tyrosine and tryptophan biosynthesis	hsa00400	compound	4	1	0	2	2
Arginine biosynthesis	hsa00220	enzyme	13	3	3	0	10
Alanine, aspartate and glutamate metabolism	hsa00250	enzyme	30	11	3	16	11
Glycine, serine and threonine metabolism	hsa00260	enzyme	30	5	1	17	12
Cysteine and methionine metabolism	hsa00270	enzyme	32	10	0	23	9
Valine, leucine and isoleucine degradation	hsa00280	enzyme	40	6	3	11	26
Lysine degradation	hsa00310	enzyme	19	5	5	0	14
Arginine and proline metabolism	hsa00330	enzyme	36	9	4	10	22
Histidine metabolism	hsa00340	enzyme	13	4	0	7	6
Tyrosine metabolism	hsa00350	enzyme	39	12	1	18	20
Phenylalanine metabolism	hsa00360	enzyme	8	3	0	6	2
Tryptophan metabolism	hsa00380	enzyme	37	16	4	21	12
Phenylalanine, tyrosine and tryptophan biosynthesis	hsa00400	enzyme	5	1	1	0	4
**1.6 Metabolism of other amino acids**							
beta-Alanine metabolism	hsa00410	compound	21	10	4	11	6
Taurine and hypotaurine metabolism	hsa00430	compound	8	4	3	2	3
Phosphonate and phosphinate metabolism	hsa00440	compound	6	2	2	0	4
Selenocompound metabolism	hsa00450	compound	20	8	7	2	11
D-Glutamine and D-glutamate metabolism	hsa00471	compound	6	2	2	0	4
D-Arginine and D-ornithine metabolism	hsa00472	compound	4	2	2	0	2
Glutathione metabolism	hsa00480	compound	28	8	6	9	13
beta-Alanine metabolism	hsa00410	enzyme	18	4	3	2	13
Taurine and hypotaurine metabolism	hsa00430	enzyme	5	3	2	2	1
Phosphonate and phosphinate metabolism	hsa00440	enzyme	4	2	2	0	2
Selenocompound metabolism	hsa00450	enzyme	9	5	4	2	3
D-Glutamine and D-glutamate metabolism	hsa00471	enzyme	4	2	2	0	2
Glutathione metabolism	hsa00480	enzyme	23	8	1	9	13
**1.7 Glycan biosynthesis and metabolism**							
N-Glycan biosynthesis	hsa00510	compound	39	9	6	5	28
Mucin type O-glycan biosynthesis	hsa00512	compound	12	6	2	6	4

Mannose type O-glycan biosynthesis	hsa00515	compound	8	4	3	2	3
Glycosaminoglycan degradation	hsa00531	compound	23	5	4	2	17
Glycosaminoglycan biosynthesis - chondroitin sulfate / dermatan sulfate	hsa00532	compound	10	2	2	0	8
Glycosaminoglycan biosynthesis - heparan sulfate / heparin	hsa00534	compound	4	1	1	0	3
Glycosylphosphatidylinositol (GPI)-anchor biosynthesis	hsa00563	compound	14	5	5	0	9
Glycosphingolipid biosynthesis - lacto and neolacto series	hsa00601	compound	48	23	6	24	18
Glycosphingolipid biosynthesis - globo and isoglobo series	hsa00603	compound	15	4	0	10	5
Glycosphingolipid biosynthesis - ganglio series	hsa00604	compound	21	7	1	14	6
N-Glycan biosynthesis	hsa00510	enzyme	33	5	1	8	24
Mucin type O-glycan biosynthesis	hsa00512	enzyme	10	5	1	6	3
Mannose type O-glycan biosynthesis	hsa00515	enzyme	4	2	1	3	0
Glycosaminoglycan degradation	hsa00531	enzyme	20	7	5	4	11
Glycosaminoglycan biosynthesis - chondroitin sulfate / dermatan sulfate	hsa00532	enzyme	7	1	1	0	6
Glycosaminoglycan biosynthesis - heparan sulfate / heparin	hsa00534	enzyme	3	1	1	0	2
Glycosylphosphatidylinositol (GPI)-anchor biosynthesis	hsa00563	enzyme	9	2	1	2	6
Glycosphingolipid biosynthesis - lacto and neolacto series	hsa00601	enzyme	43	23	5	28	10
Glycosphingolipid biosynthesis - globo and isoglobo series	hsa00603	enzyme	15	1	0	15	0
Glycosphingolipid biosynthesis - ganglio series	hsa00604	enzyme	22	7	2	10	10
**1.8 Metabolism of cofactors and vitamins**							
Ubiquinone and other terpenoid-quinone biosynthesis	hsa00130	compound	9	3	1	6	2
One carbon pool by folate	hsa00670	compound	9	2	0	5	4
Thiamine metabolism	hsa00730	compound	7	3	2	2	3
Riboflavin metabolism	hsa00740	compound	4	1	0	2	2
Vitamin B6 metabolism	hsa00750	compound	9	2	0	5	4
Nicotinate and nicotinamide metabolism	hsa00760	compound	15	3	2	2	11
Pantothenate and CoA biosynthesis	hsa00770	compound	19	7	6	2	11
Biotin metabolism	hsa00780	compound	10	5	4	2	4
Lipoic acid metabolism	hsa00785	compound	5	3	2	2	1
Folate biosynthesis	hsa00790	compound	27	8	4	7	16
Retinol metabolism	hsa00830	compound	18	9	3	9	6
Porphyrin and chlorophyll metabolism	hsa00860	compound	30	10	6	6	18
Ubiquinone and other terpenoid-quinone biosynthesis	hsa00130	enzyme	8	3	1	6	1
One carbon pool by folate	hsa00670	enzyme	22	1	0	2	20
Thiamine metabolism	hsa00730	enzyme	5	1	0	5	0
Riboflavin metabolism	hsa00740	enzyme	5	1	1	0	4

Vitamin B6 metabolism	hsa00750	enzyme	11	3	0	10	1
Nicotinate and nicotinamide metabolism	hsa00760	enzyme	21	4	1	9	11
Pantothenate and CoA biosynthesis	hsa00770	enzyme	13	4	4	0	9
Biotin metabolism	hsa00780	enzyme	3	1	1	0	2
Lipoic acid metabolism	hsa00785	enzyme	4	2	2	0	2
Folate biosynthesis	hsa00790	enzyme	32	9	5	6	21
Retinol metabolism	hsa00830	enzyme	20	11	3	17	0
Porphyrin and chlorophyll metabolism	hsa00860	enzyme	18	5	1	7	10
**1.9 Metabolism of terpenoids and polyketides**							
Terpenoid backbone biosynthesis	hsa00900	compound	18	5	1	7	10
Terpenoid backbone biosynthesis	hsa00900	enzyme	15	4	1	6	8
**1.10 Biosynthesis of other secondary metabolites**							
Caffeine metabolism	hsa00232	compound	10	5	3	3	4
Neomycin, kanamycin and gentamicin biosynthesis	hsa00524	compound	2	1	1	0	1
Caffeine metabolism	hsa00232	enzyme	6	4	1	4	1
**1.11 Xenobiotics biodegradation and metabolism**							
Metabolism of xenobiotics by cytochrome P450	hsa00980	compound	68	33	15	30	23
Drug metabolism - cytochrome P450	hsa00982	compound	55	30	15	25	15
Drug metabolism - other enzymes	hsa00983	compound	39	14	8	10	21
Metabolism of xenobiotics by cytochrome P450	hsa00980	enzyme	39	20	15	10	14
Drug metabolism - cytochrome P450	hsa00982	enzyme	23	13	9	7	7
Drug metabolism - other enzymes	hsa00983	enzyme	38	13	10	8	20
